# Association of cardiovascular-kidney-metabolic syndrome with depression and all-cause mortality: a population-based observational study

**DOI:** 10.3389/fnut.2025.1620008

**Published:** 2025-11-03

**Authors:** Wei Liu, Zhijuan Li, Pu Ma

**Affiliations:** Department of Cardiovascular, The First Affiliated Hospital, and College of Clinical Medicine of Henan University of Science and Technology, Luoyang, China

**Keywords:** cardiovascular-kidney-metabolic syndrome, depression, mortality, National Health and Nutrition Examination Survey, observational study

## Abstract

**Background:**

Cardiovascular-Kidney-Metabolic Syndrome (CKM) is a novel multi-system disease defined by the American Heart Association (AHA). This study aims to investigate the prevalence of depression in different stages of CKM syndrome and its relationship with the risk of all-cause mortality in patients with depression.

**Methods:**

The data used for this study were sourced from the National Health and Nutrition Examination Survey (NHANES) and the National Death Index (NDI) database, which were conducted from 2005 to 2020. CKM syndrome was classified into stages 0–4, among which stages 3–4 were defined as advanced CKM syndrome. Depression was defined according to the Patient Health Questionnaire - 9 (PHQ-9). Multivariable logistic regression and multivariable Cox regression were performed as the main analytical methods. Age-standardized prevalence analysis among four groups and Kaplan–Meier survival analysis were also carried out. Finally, a stratified analysis was conducted.

**Results:**

A total of 15,156 participants were included in this study, with a median age of 49 years, among whom 7,608 (50%) were males. The numbers of participants in stages 0–4 of CKM were 1,360, 3,029, 8,246, 842, and 1,679, respectively. The age-standardized prevalence rates of depression in each stage were 3.40, 5.63, 7.44, 16.48, and 18.49%, respectively. In the process of logistic regression, all confounding variables were adjusted for comparison with the participants in stage 0 of CKM syndrome. It was revealed that the prevalence rates of depression in the participants of stage 1, stage 2, and stage 3 did not increase significantly (all *p* > 0.05), while the prevalence rate of depression in the population of stage 4 increased significantly (OR: 1.98, 95%CI: 1.40–2.82, *p* < 0.001). With stages 3–4 further defined as advanced CKM syndrome, the likelihood of depression in advanced CKM stages was found higher (OR: 1.47, 95%CI: 1.24–1.76, *p* < 0.0001). In addition, a total of 1,037 participants with depression were included for the survival analysis, and the advanced CKM stages significantly increased the risk of all-cause mortality in these patients (HR: 1.94, 95%CI: 1.29–2.93, *p* = 0.002). The subgroup analysis showed that the correlation between CKM syndrome and depression was stronger in the younger group (<60 years).

**Conclusion:**

Our study shows that the risk of depression increases across stages 0–4 of CKM syndrome. The prevalence of depression in advanced CKM syndrome is also significantly elevated. Moreover, advanced CKM syndrome further increases the risk of all-cause mortality in patients with depression. These findings suggest that medical workers should be vigilant about the mental status of patients with advanced CKM syndrome and the risk of poor prognosis when it is complicated by depression.

## Introduction

Depression is a mental disorder, also known as depressive disorder and mainly manifested as persistent low mood. Some patients with depression exhibit self-harm and suicidal behaviors and are possibly accompanied by various psychotic symptoms such as delusions and hallucinations (1). Among various disabling mental disorders, depression and anxiety disorder rank among the top two. According to the data released by the World Health Organization (WHO), depression has a quite widespread impact globally, affecting approximately 5% of the world population. Moreover, relevant predictions indicate that by 2030, depression will rise to the top of the global disease burden, exerting non-negligible pressure on human health and social development (2–4). As for the general population in the United States, the prevalence rate of depression within a 12-month period is close to 7%, and detailed analysis has revealed the significant differences observed in terms of age where the probability of young people aged 18 to 29 suffering from depression is more than twice as high as that of the elderly population aged 60 and above. At the same time, when analyzed from the perspective of gender, the female population is more vulnerable to depression since the initial stage of puberty, with the prevalence rate being 0.5 to 2 times higher compared with men (5). Depression has a negative impact on the life, work performance and even interpersonal relationships of an individual. In severe cases, it can also lead to an increase in the suicide rate, which undoubtedly imposes a heavy burden and great pressure on the specialized psychiatric medical services provided by the government. In view of this severe situation, it is imperative to conduct a comprehensive and in-depth analysis and discussion on the issue of depression for exploring the practical and effective coping strategies and for alleviating its many adverse effects.

In 2023, the American Heart Association (AHA) proposed the concept of Cardiovascular–kidney–metabolic (CKM) syndrome (6). CKM syndrome falls within the category of systemic diseases, and the core of its pathogenesis lies in the interaction among metabolic risk factors, chronic kidney disease (CKD), and the cardiovascular system. This profound mutual influence further triggers the dysfunction of multiple organs, ultimately resulting in a consistently high incidence of adverse cardiovascular events (7). From an individual perspective, once metabolic abnormalities, cardiovascular diseases (CVD), and CKD are intricately intertwined and manifest simultaneously, the prognosis of that individual is highly likely to deteriorate sharply. According to the severity of the condition, CKM syndrome can be meticulously classified into five levels ranging from 0 to 4, among which levels 3 and 4 are in the late stage of the disease. There is conclusive data indicating that among the adult population of the United States, nearly 90% of people have already met the diagnostic criteria for stage 1 of CKM syndrome, and the proportion of adults who meet the diagnostic criteria for the late stage (that is, stages 3–4) is approximately 15% (8). Previous studies have shown that metabolic factors, CKD, and CVD are all risk factors for depression (9, 10). However, the prevalence of depression across different stages of CKM remains to be revealed. In addition, Zhu et al. (11) indicated that compared with stage 0, stages 3–4 (but not stages 1–2) of CKM syndrome are associated with an increased risk of premature death. Importantly, the positive correlation between depressive symptoms and all-cause mortality persists across different CKM stages (12). Nevertheless, in patients with depression, it remains unknown how the prognosis of all-cause mortality changes with the progression of CKM syndrome.

Against this backdrop, this study aims to explore the associations between CKM syndrome, depression, and the risk of all-cause mortality in the U.S. adult population using the data sourced from the National Health and Nutrition Examination Survey (NHANES). It is hypothesized that higher stages of CKM syndrome increase the prevalence of depression and contribute to the poor prognosis in patients with depression. By clarifying this relationship, our goal is to provide a scientific basis for the early identification, risk stratification, and development of intervention strategies for comorbid CKM syndrome and depression. This is expected to reduce the risk of depression and improve the prognosis of depressed patients in clinical practice by managing CKM-related metabolic abnormalities. Meanwhile, it can provide theoretical support for the formulation of joint prevention and control measures intended to combat mental health and chronic metabolic diseases for the U.S. adult population, with the ultimate aim to alleviate the social and disease burdens caused by depression and all-cause mortality.

## Methods

### The design of this study

NHANES selects representative residents across the United States through stratified multistage sampling. Firstly, computer-assisted interviews are conducted in households to collect information on demographics, health behaviors, and diet. Then, standardized physical examinations including measurements of height, weight, blood pressure, dentistry, vision, and collection of laboratory specimens are completed in the Mobile Examination Center (MEC). All measurements follow the operation manuals compiled by the Centers for Disease Control and Prevention (CDC). Instruments are calibrated according to the specified cycle, and gold standard checks are carried out regularly to ensure consistency. Interviewers and physical examination technicians are required to complete a full set of trainings covering interview skills, role-playing, cultural sensitivity, instrument use, and calibration. Deviations are continuously monitored and corrected through multi-level quality control measures such as on-site supervision, random spot checks, and retraining (13). For more information, please refer to https://www.cdc.gov/nhanes.

This study combines a cross-sectional study with a longitudinal cohort study. The investigation into the relationship between CKM syndrome and depression was conducted through a cross-sectional design, while the association between CKM syndrome and the risk of all-cause mortality in patients with depression was explored through a longitudinal cohort design.

### Participants

**Figure 1:**
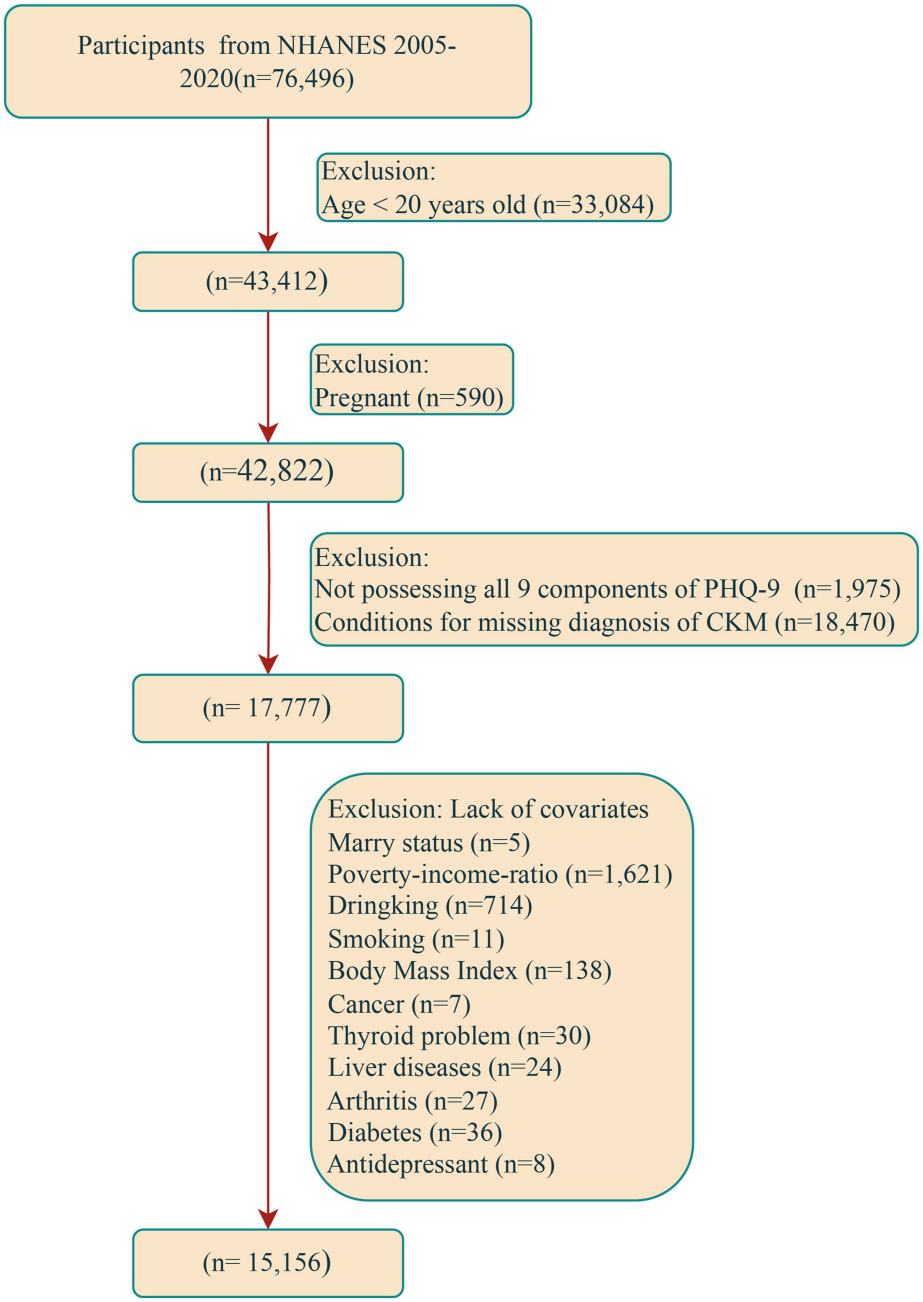
Flow chart of participant recruitment. NHANES, National Health and Nutrition Examination Survey; CKM, cardiovascular-kidney-metabolic; PHQ-9, Patient Health Questionnaire-9.

NHANES is a continuous national cross-sectional survey project conducted every two years among the American population and through varius means such as personal interviews, standardized questionnaires, and physical examinations. This survey collects the participants’ basic demographic data, socio-economic background information, as well as the data related to health and nutritional status. The Ethics Review Committee of the National Center for Health Statistics in the United States has approved all NHANES survey protocols and every individual participating in the survey has signed a written informed consent form. The data used for this study were sourced from 8 non-overlapping cycles of NHANES during the period from 2005 to 2020, with the specific inclusion and exclusion steps referred to in Figure 1. During this time period, a total of 76,496 people participated in the survey. Successively, 33,084 individuals under the age of 20, 590 pregnant women, 1,975 participants without the complete 9 items of the Patient Health Questionnaire-9 (PHQ-9), 18,470 participants lacking the diagnostic criteria for CKM, and 2,621 participants lacking necessary covariables were excluded. Finally, the sample size of this study was 15,156.

### CKM syndrome and advanced CKM syndrome (exposure)

Recently, the AHA and Aggarwal et al. have proposed the concept of CKM syndrome (6, 8). According to the progression of the condition, this syndrome is meticulously divided into five stages, namely stages 0 to 4. Specifically, stage 0 is determined when both the body mass index (BMI) and waist circumference are within the normal range, with the participants meeting this condition not included in higher stages. Once a participant shows an increase in BMI, an increase in waist circumference, or the features of prediabetes, he or she is classified into stage 1. When a participant has metabolic-related risk factors or is at a moderate or high risk of developing CKD, he or she is classified into stage 2. The criterion used to define stage 3 is that the participant has a high risk of CKD or is predicted to have a high risk of developing CVD according to the 10-year CVD prediction model. If a participant has already suffered from CVD, then he or she is classified into stage 4 (11). Notably, the determination of the risk of developing CKD is based on the standards established by the Kidney Disease Improving Global Outcomes (KDIGO) (14), while the risk of developing CVD is estimated through the Prediction of Cardiovascular Events (PREVENT) equation (15). More detailed information can be found in Supplementary Table 1. According to previous relevant research findings, it is customary to define stages 3–4 as advanced CKM syndrome (16).

### Depression (outcome)

As a widely used tool for screening and evaluating depression in clinical practice and research, PHQ-9 was applied in this study to assess the depressive symptoms of subjects within the recent half month (17). The PHQ-9 consists of 9 sub-items, each of which is classified into four grades ranging from 0 to 3 points. By summing up the scores of all items, a total score ranging from 0 to 27 can be obtained. In this study, a total score of PHQ-9 ≥ 10 was defined as depression. This cut-off point is frequently used in clinical and epidemiological studies to define depression and has been clinically validated with a sensitivity of 88% and a specificity of 88% (17–19).

### Determination of survival status (outcome)

In the United States, the mortality status of an individual is meticulously recorded by the National Death Index (NDI). In this study, the research team precisely matched these participants with the NDI database to accurately determine the survival status of each participant in the NHANES project. Specifically, when the matching operation is successfully completed, the corresponding individual is firmly identified as deceased, with the exact date of death carefully recorded. Conversely, if the matching fails, it means that the individual remains alive. For such surviving individuals, December 31, 2019 is set as the end time point of their follow-up (20, 21). As for the duration of the follow-up, it is measured in years and determined by calculating the difference between the date of death (for deceased individuals) or the end date of the follow-up (for surviving individuals) and the date when the individual was initially recruited.

### Covariables

In the process of research, confounding factors were determined in two ways. On the one hand, plenty of previous relevant literature materials were extensively and deeply studied. On the other hand, the unique characteristics of the NHANES database itself were fully combined to accurately identify the confounding factors (4, 22, 23). The specific data collection covers multiple dimensions. At the demographic and socio-economic level, various data such as gender (presented as a binary classification, namely male and female), age (recorded as a continuous value), race (subdivided into five categories), educational background (divided into two categories), marital status (classified into three categories), and poverty income ratio (PIR, also divided into three categories) were collected. Regarding lifestyle and physical indicators, such information as smoking status (divided into three categories), alcohol consumption status (refined into five categories), physical activity level (divided into four categories), total dietary energy intake (set as three categories), and body mass index (BMI, divided into four categories) was collected. In addition, health-related characteristics were also taken into consideration, such as cancer (binary classification: with or without cancer), thyroid problems (binary: present or absent), arthritis (also binary: with or without the disease), liver disease (also binary: with or without the disease), diabetes (divided into four categories), hypertension (binary: with or without the disease), and the use of antidepressant medications (divided into three categories). For more detailed data presentation, please refer to Table 1.

**Table 1:** Baseline characteristics based on the stages of CKM syndrome in the cross-sectional study.

Characteristics	Total (*n* = 15,156)	CKM 0 (*n* = 1,360)	CKM 1 (*n* = 3,029)	CKM 2 (*n* = 8,246)	CKM 3 (*n* = 842)	CKM 4 (*n* = 1,679)	*P*-value
Depression, *n* (%)							< 0.001
No	13,883 (92)	1,285 (94)	2,851 (94)	7,547 (92)	783 (93)	1,417 (84)	
Yes	1,273 (8)	75 (6)	178 (6)	699 (8)	59 (7)	262 (16)	
Age (Year), Median (Q1, Q3)	49 (35, 64)	31 (24, 42)	38 (28, 50)	51 (38, 62)	78 (72, 80)	68 (58, 76.5)	< 0.001
Sex, *n* (%)							< 0.001
Female	7,548 (50)	844 (62)	1,596 (53)	4,078 (49)	303 (36)	727 (43)	
Male	7,608 (50)	516 (38)	1,433 (47)	4,168 (51)	539 (64)	952 (57)	
Race, *n* (%)							< 0.001
Mexican American	2,225 (15)	144 (11)	514 (17)	1,335 (16)	86 (10)	146 (9)	
Non-Hispanic Black	3,246 (21)	212 (16)	622 (21)	1863 (23)	168 (20)	381 (23)	
Non-Hispanic White	6,699 (44)	692 (51)	1,208 (40)	3,395 (41)	475 (56)	929 (55)	
Other Hispanic	1,390 (9)	107 (8)	309 (10)	781 (9)	62 (7)	131 (8)	
Other Race - Including Multi-Racial	1,596 (11)	205 (15)	376 (12)	872 (11)	51 (6)	92 (5)	
Education level, *n* (%)							< 0.001
No college	6,949 (46)	427 (31)	1,157 (38)	3,904 (47)	520 (62)	941 (56)	
College or equivalent	8,207 (54)	933 (69)	1872 (62)	4,342 (53)	322 (38)	738 (44)	
Marital status, *n* (%)							< 0.001
No married	2,753 (18)	506 (37)	756 (25)	1,353 (16)	25 (3)	113 (7)	
Divorced or separated or widowed	2,790 (18)	112 (8)	339 (11)	1,528 (19)	284 (34)	527 (31)	
Already married or cohabitation	9,613 (63)	742 (55)	1934 (64)	5,365 (65)	533 (63)	1,039 (62)	
PIR, *n* (%)							< 0.001
<1.3	4,601 (30)	362 (27)	834 (28)	2,518 (31)	285 (34)	602 (36)	
1.3–3.5	5,802 (38)	481 (35)	1,123 (37)	3,122 (38)	384 (46)	692 (41)	
>3.5	4,753 (31)	517 (38)	1,072 (35)	2,606 (32)	173 (21)	385 (23)	
Smoking status, *n* (%)							< 0.001
Never smoked	8,250 (54)	895 (66)	1857 (61)	4,461 (54)	374 (44)	663 (39)	
Former smoker	3,746 (25)	171 (13)	587 (19)	2016 (24)	337 (40)	635 (38)	
Current smoker	3,160 (21)	294 (22)	585 (19)	1769 (21)	131 (16)	381 (23)	
Drinking status, *n* (%)							< 0.001
Former drinker	2,312 (15)	92 (7)	282 (9)	1,220 (15)	251 (30)	467 (28)	
Current heavy drinker	3,142 (21)	330 (24)	742 (24)	1823 (22)	45 (5)	202 (12)	
Current mild drinker	5,342 (35)	491 (36)	1,085 (36)	2,817 (34)	333 (40)	616 (37)	
Current moderate drinker	2,366 (16)	277 (20)	586 (19)	1,281 (16)	50 (6)	172 (10)	
Never drinked	1994 (13)	170 (12)	334 (11)	1,105 (13)	163 (19)	222 (13)	
Energy intake, *n* (%)							0.002
Low intake	6,213 (41)	517 (38)	1,220 (40)	3,485 (42)	329 (39)	662 (39)	
High intake	6,145 (41)	561 (41)	1,268 (42)	3,276 (40)	327 (39)	713 (42)	
Unknown	2,798 (18)	282 (21)	541 (18)	1,485 (18)	186 (22)	304 (18)	
Physical activity [MET, minutes/week, *n* (%)]							< 0.001
<700	2,920 (19)	259 (19)	502 (17)	1,665 (20)	172 (20)	322 (19)	
700–2,400	3,437 (23)	377 (28)	743 (25)	1832 (22)	152 (18)	333 (20)	
> = 2,400	5,079 (34)	549 (40)	1,248 (41)	2,773 (34)	142 (17)	367 (22)	
Not report	3,720 (25)	175 (13)	536 (18)	1976 (24)	376 (45)	657 (39)	
BMI (Kg/m^2^), *n* (%)							< 0.001
18.5–25	4,165 (27)	1,257 (92)	686 (23)	1,655 (20)	231 (27)	336 (20)	
<18.5	243 (2)	103 (8)	28 (1)	75 (1)	13 (2)	24 (1)	
25–30	4,985 (33)	0 (0)	1,432 (47)	2,676 (32)	330 (39)	547 (33)	
> = 30	5,763 (38)	0 (0)	883 (29)	3,840 (47)	268 (32)	772 (46)	
Cancer, *n* (%)							< 0.001
No	13,716 (90)	1,297 (95)	2,892 (95)	7,549 (92)	646 (77)	1,332 (79)	
Yes	1,440 (10)	63 (5)	137 (5)	697 (8)	196 (23)	347 (21)	
Thyroid problem, *n* (%)							< 0.001
No	13,601 (90)	1,281 (94)	2,822 (93)	7,427 (90)	710 (84)	1,361 (81)	
Yes	1,555 (10)	79 (6)	207 (7)	819 (10)	132 (16)	318 (19)	
Liver problem, *n* (%)							< 0.001
No	14,543 (96)	1,338 (98)	2,964 (98)	7,881 (96)	802 (95)	1,558 (93)	
Yes	613 (4)	22 (2)	65 (2)	365 (4)	40 (5)	121 (7)	
Arthritis, *n* (%)							< 0.001
No	10,952 (72)	1,235 (91)	2,606 (86)	5,898 (72)	459 (55)	754 (45)	
Yes	4,204 (28)	125 (9)	423 (14)	2,348 (28)	383 (45)	925 (55)	
DM, *n* (%)							< 0.001
DM	3,136 (21)	0 (0)	0 (0)	1909 (23)	484 (57)	743 (44)	
IFG	1,446 (10)	0 (0)	223 (7)	971 (12)	76 (9)	176 (10)	
IGT	1,121 (7)	35 (3)	143 (5)	730 (9)	84 (10)	129 (8)	
No	9,453 (62)	1,325 (97)	2,663 (88)	4,636 (56)	198 (24)	631 (38)	
Hypertension, *n* (%)							< 0.001
No	8,645 (57)	1,360 (100)	3,029 (100)	3,709 (45)	169 (20)	378 (23)	
Yes	6,511 (43)	0 (0)	0 (0)	4,537 (55)	673 (80)	1,301 (77)	
Antidepressant, *n* (%)							< 0.001
No	6,313 (42)	877 (64)	1990 (66)	3,228 (39)	99 (12)	119 (7)	
Other drugs	7,148 (47)	389 (29)	826 (27)	4,057 (49)	657 (78)	1,219 (73)	
Yes	1,695 (11)	94 (7)	213 (7)	961 (12)	86 (10)	341 (20)	

CKM syndrome, cardiovascular-kidney-metabolic syndrome; PIR, poverty-to-income ratio; MET, metabolic equivalent; DM, diabetes mellitus; IFG, impaired fasting glycaemia; IGT, impaired glucose tolerance.

### Statistical analysis

When describing the characteristics of the participants, the Kruskal-Wallis test was used for the intergroup comparison of continuous variables, and the results were presented as median (interquartile range, IQR); the chi-square test was applied for categorical variables, which were expressed as the number of cases (n) and percentage (%). To describe the actual prevalence of depression among different CKM syndrome groups, the age-standardized estimated prevalence of depression and its 95% confidence intervals (CIs) in each CKM syndrome stage were calculated. To more accurately quantify the strength of the independent association between CKM syndrome and depression and explore the stability of this association under different classification methods, CKM syndrome was included in the multivariable logistic regression model as a five-category variable (stages 0–4) or a binary variable (advanced CKM syndrome/non-advanced CKM syndrome) to estimate the odds ratios (ORs) and their 95% CIs for the association between CKM syndrome and depression. To investigate the relationship between CKM syndrome and the prognosis of patients with depression, survival analysis was performed. Firstly, Kaplan–Meier survival analysis and the log-rank test were used to intuitively describe and compare the survival status across different CKM syndrome stages and quantify the distribution characteristics of survival time. In addition, we further accurately quantified the strength of the independent association between CKM syndrome and the prognosis of patients with depression. Due to the limitation of sample size, CKM syndrome was included in the multivariable Cox proportional hazards model as a binary variable (advanced CKM syndrome/non-advanced CKM syndrome) to estimate the hazard ratios (HRs) and their 95% CIs for the independent association between CKM syndrome and the risk of all-cause mortality in patients with depression. To verify the robustness of the association, multiple models were constructed in this study: Model 0 was unadjusted for any covariates; Model 1 was adjusted for gender, age, race, educational level, marital status, and family economic status; Model 2 was further adjusted for alcohol consumption, smoking, exercise intensity, dietary energy intake, and BMI; Model 3 was further adjusted for comorbidities and a history of antidepressant use. To ensure the reliability, validity, and interpretability of the regression analysis results, the variance inflation factor (VIF) was used to assess multicollinearity, and all VIF values of variables in this study were less than 10. The Schoenfeld residuals were used to test the assumption of the Cox proportional hazards model, and this prerequisite assumption was satisfied in this study. Subgroup analysis was conducted, and the likelihood ratio test was used to explore the interaction between covariates and CKM syndrome. To verify the robustness of the results, three sensitivity analyses were performed: first, the sampling weight of the sample was considered, and “wtmec2yr” was selected as the weight since multiple variables used in this study were obtained from laboratory tests; second, mild depression (PHQ-9 score of 5–9) was also defined as depression; third, multiple imputation was performed for missing covariates.

All the statistical procedures involved in this study were completed using R software version 4.3.3 (R Foundation for Statistical Computing). In the present study, statistical significance was defined as a two-tailed *p*-value less than 0.05.

## Results

### Population characteristics

According to the research design, the participants were grouped by their CKM syndrome stages, with the corresponding baseline characteristics presented in Table 1. After strict screening, a total of 15,156 samples were included in this study, with a median age of 49 years, and 7,608 (50%) were males. A total of 1,273 participants were diagnosed with depression. The numbers of participants in CKM stages 0–4 were 1,360, 3,029, 8,246, 842, and 1,679, respectively; the prevalence rates of depression in these five stages were 6, 6, 8, 7, and 16%, respectively (*p* < 0.001). Compared with the participants in stage 0 of CKM syndrome, those in stage 4 were more likely to be older male non-Hispanic blacks. Also, they were more likely to be divorced people with low education levels and in poverty. At the same time, they were more likely to have a history of alcohol consumption and smoking, and to exercise less. Moreover, compared with the participants in stage 0 of CKM syndrome, those in stage 4 were more likely to have various comorbidities such as cancer, thyroid problems, liver problems, arthritis, diabetes, and hypertension. Finally, the proportion of individuals in stage 4 of CKM syndrome using antidepressant medications was also significantly higher.

In the process of survival analysis, 236 out of 1,273 participants with depression were lost to follow-up during the median follow-up period of 8.08 years, with 1,037 participants finally included. Compared with the survivor group, the proportion of those in the advanced CKM syndrome stage in the death group increased by approximately twice (21% vs. 60%). The death group was more likely to be older male non-Hispanic whites, divorced people with low education levels. At the same time, the death group was more likely to have a history of alcohol consumption, low dietary energy intake, and less exercise. Finally, the death group was also more likely to have various comorbidities such as cancer, thyroid problems, diabetes, hypertension, and to use antidepressant medications (Table 2).

**Table 2:** Baseline characteristics based on the survival condition of the participants with depression in survival analysis.

Characteristics	Total (*n* = 1,037)	Alive (*n* = 893)	All-cause mortality (*n* = 144)	*P*-value
Follow-up duration, Median (Q1, Q3)	8.08 (5.33, 10.92)	8.67 (5.92, 11.17)	4.96 (2.31, 7.42)	< 0.001
CKM, *n* (%)				< 0.001
Non-advanced CKM syndrome	760 (73)	702 (79)	58 (40)	
Advanced CKM syndrome	277 (27)	191 (21)	86 (60)	
Age (Year), Median (Q1, Q3)	51 (38, 62)	49 (36, 60)	63 (55, 73)	< 0.001
Sex, *n* (%)				0.018
Female	657 (63)	579 (65)	78 (54)	
Male	380 (37)	314 (35)	66 (46)	
Race, *n* (%)				< 0.001
Mexican American	153 (15)	141 (16)	12 (8)	
Non-Hispanic Black	225 (22)	192 (22)	33 (23)	
Non-Hispanic White	476 (46)	389 (44)	87 (60)	
Other Hispanic	126 (12)	119 (13)	7 (5)	
Other Race - Including Multi-Racial	57 (5)	52 (6)	5 (3)	
Education level, *n* (%)				0.034
No college	641 (62)	540 (60)	101 (70)	
College or equivalent	396 (38)	353 (40)	43 (30)	
Marital status, *n* (%)				< 0.001
Divorced or separated or widowed	379 (37)	303 (34)	76 (53)	
No married	204 (20)	192 (22)	12 (8)	
Already married or cohabitation	454 (44)	398 (45)	56 (39)	
PIR, *n* (%)				0.152
<1.3	595 (57)	505 (57)	90 (62)	
1.3–3.5	314 (30)	271 (30)	43 (30)	
>3.5	128 (12)	117 (13)	11 (8)	
Smoking status, *n* (%)				0.177
Never smoked	412 (40)	362 (41)	50 (35)	
Former smoker	229 (22)	200 (22)	29 (20)	
Current smoker	396 (38)	331 (37)	65 (45)	
Drinking status, *n* (%)				0.011
Former drinker	236 (23)	189 (21)	47 (33)	
Current heavy drinker	252 (24)	223 (25)	29 (20)	
Current mild drinker	258 (25)	223 (25)	35 (24)	
Current moderate drinker	142 (14)	131 (15)	11 (8)	
Never drinked	149 (14)	127 (14)	22 (15)	
Energy intake, *n* (%)				0.003
Low intake	442 (43)	383 (43)	59 (41)	
High intake	426 (41)	378 (42)	48 (33)	
Unknown	169 (16)	132 (15)	37 (26)	
Physical activity [MET, minutes/week, *n* (%)]				< 0.001
<700	223 (22)	191 (21)	32 (22)	
700–2,400	177 (17)	161 (18)	16 (11)	
> = 2,400	257 (25)	244 (27)	13 (9)	
Not report	380 (37)	297 (33)	83 (58)	
BMI (Kg/m^2^), *n* (%)				0.206
18.5–25	245 (24)	204 (23)	41 (28)	
<18.5	21 (2)	16 (2)	5 (3)	
25–30	262 (25)	230 (26)	32 (22)	
> = 30	509 (49)	443 (50)	66 (46)	
Cancer, *n* (%)				< 0.001
No	926 (89)	812 (91)	114 (79)	
Yes	111 (11)	81 (9)	30 (21)	
Thyroid problem, *n* (%)				0.848
No	862 (83)	741 (83)	121 (84)	
Yes	175 (17)	152 (17)	23 (16)	
Liver problem, *n* (%)				0.061
No	958 (92)	831 (93)	127 (88)	
Yes	79 (8)	62 (7)	17 (12)	
Arthritis, *n* (%)				< 0.001
No	537 (52)	491 (55)	46 (32)	
Yes	500 (48)	402 (45)	98 (68)	
DM, *n* (%)				< 0.001
DM	305 (29)	232 (26)	73 (51)	
IFG	71 (7)	56 (6)	15 (10)	
IGT	96 (9)	82 (9)	14 (10)	
No	565 (54)	523 (59)	42 (29)	
Hypertension, *n* (%)				< 0.001
No	482 (46)	444 (50)	38 (26)	
Yes	555 (54)	449 (50)	106 (74)	
Antidepressant, *n* (%)				< 0.001
No	273 (26)	257 (29)	16 (11)	
Other drugs	416 (40)	342 (38)	74 (51)	
Yes	348 (34)	294 (33)	54 (38)	

CKM syndrome, cardiovascular-kidney-metabolic syndrome; PIR, poverty-to-income ratio; MET, metabolic equivalent; DM, diabetes mellitus; IFG, impaired fasting glycaemia; IGT, impaired glucose tolerance.

### Estimation of the association between CKM syndrome and depression

**Figure 2:**
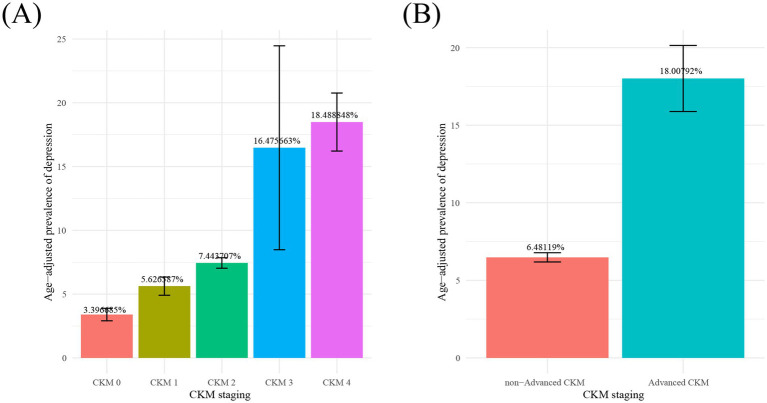
Age-adjusted prevalence of depression in different stages of CKM syndrome from 15,156 participants in cross-sectional study. Notes: Numbers at the top of the bars represent the percentage. Bar whiskers represent the 95% confidence level. CKM syndrome; cardiovascular-kidney-metabolic syndrome.

Figure 2 shows the age-standardized prevalence of depression among different stages of CKM syndrome. As shown in Figure 2A, the age-standardized prevalence rates of depression in stages 0–4 of CKM syndrome are 3.40, 5.63, 7.44, 16.48, and 18.49%, respectively. As shown in Figure 2B, the age-standardized prevalence rates of depression in non-advanced CKM syndrome and advanced CKM syndrome are 6.48 and 18.01%, respectively. These results suggest a probability that CKM syndrome is a risk factor for depression. When it exceeds stage 3, the prevalence rate of depression surges.

Table 3 shows the association between CKM syndrome and depression analyzed through the logistic regression model.

**Table 3:** OR estimates for the association between CKM syndrome and depression from 15,156 participants in cross-sectional study.

	Model 0		Model 1		Model 2		Model 3	
	OR (95%CI)	*P-*value	OR (95%CI)	*P-*value	OR (95%CI)	*P-*value	OR (95%CI)	*P-*value
CKM 0	Reference		Reference		Reference		Reference	
CKM 1	1.07 (0.81, 1.42)	0.63	1.17 (0.89, 1.57)	0.27	0.91 (0.67, 1.25)	0.56	1.06 (0.78, 1.45)	0.72
CKM 2	1.59 (1.25, 2.04)	<0.001	1.88 (1.46, 2.46)	<0.0001	1.36 (1.02, 1.82)	0.04	1.25 (0.93, 1.70)	0.14
CKM 3	1.29 (0.90, 1.83)	0.16	1.89 (1.26, 2.82)	0.002	1.36 (0.89, 2.07)	0.15	1.38 (0.89, 2.14)	0.15
CKM 4	3.17 (2.44, 4.16)	<0.0001	4.08 (3.01, 5.56)	<0.0001	2.74 (1.97, 3.84)	<0.0001	1.98 (1.40, 2.82)	<0.001
*p* for trend		<0.0001		<0.0001		<0.0001		<0.001
Non-Advanced CKM syndrome	Reference		Reference		Reference		Reference	
Advanced CKM syndrome	1.79 (1.56, 2.05)	<0.0001	1.94 (1.64, 2.29)	<0.0001	1.81 (1.53, 2.14)	<0.0001	1.47 (1.24, 1.76)	<0.0001

Model 0: Crude model.

Model 1: Adjusted for age, sex, race, marital status, education, and poverty-income ratio.

Model 2: Additionally adjusted for drinking, smoking, total energy intake, weekly physical activity level, and body mass index.

Model 3: further adjusted for diabetes, cancer, hypertension, liver diseases, thyroid diseases, arthritis, and antidepressant.

Non-Advanced CKM syndrome was defined as CKM syndrome in stages 0–2. Advanced CKM syndrome was defined as CKM syndrome in stages 3–4. CKM syndrome, cardiovascular-kidney-metabolic syndrome; OR, odds ratio; CI, confidence interval.

When CKM syndrome was included in the model as a five-category variable (stages 0–4), the prevalence of depression in stage 1 showed no significant change compared with stage 0 in any model. Also, the prevalence of depression in stages 2 and 3 did not increase significantly either in the fully adjusted model. Specifically, compared with stage 0, the prevalence of depression in stage 1 (OR: 1.06, 95% CI: 0.78–1.45, *p* = 0.72), stage 2 (OR: 1.25, 95% CI: 0.93–1.70, *p* = 0.14), and stage 3 (OR: 1.38, 95% CI: 0.89–2.14, *p* = 0.15) showed no significant changes in the fully adjusted model (Model 3), but the prevalence of depression in stage 4 (OR: 1.98, 95% CI: 1.40–2.82, *p* < 0.001) increased by 0.98 times. Additionally, it is worth noting that in the fully adjusted Model 3, the prevalence of depression showed an increasing trend across stages 0–4 of CKM syndrome (*P* for trend < 0.001) (Table 3).

With CKM syndrome included in the model as a binary variable of non-advanced CKM syndrome (stages 0–2) and advanced CKM syndrome (stages 3–4), the prevalence rate of depression in the advanced CKM syndrome group increased significantly in all models when compared with the reference group of stages 0–2. In the fully adjusted Model 3, the prevalence rate of depression in the advanced CKM syndrome group increased by 47% (OR: 1.47, 95% CI: 1.24–1.76, *p* < 0.0001) (Table 3).

### CKM syndrome increases the risk of all-cause mortality in depressed participants

**Figure 3:**
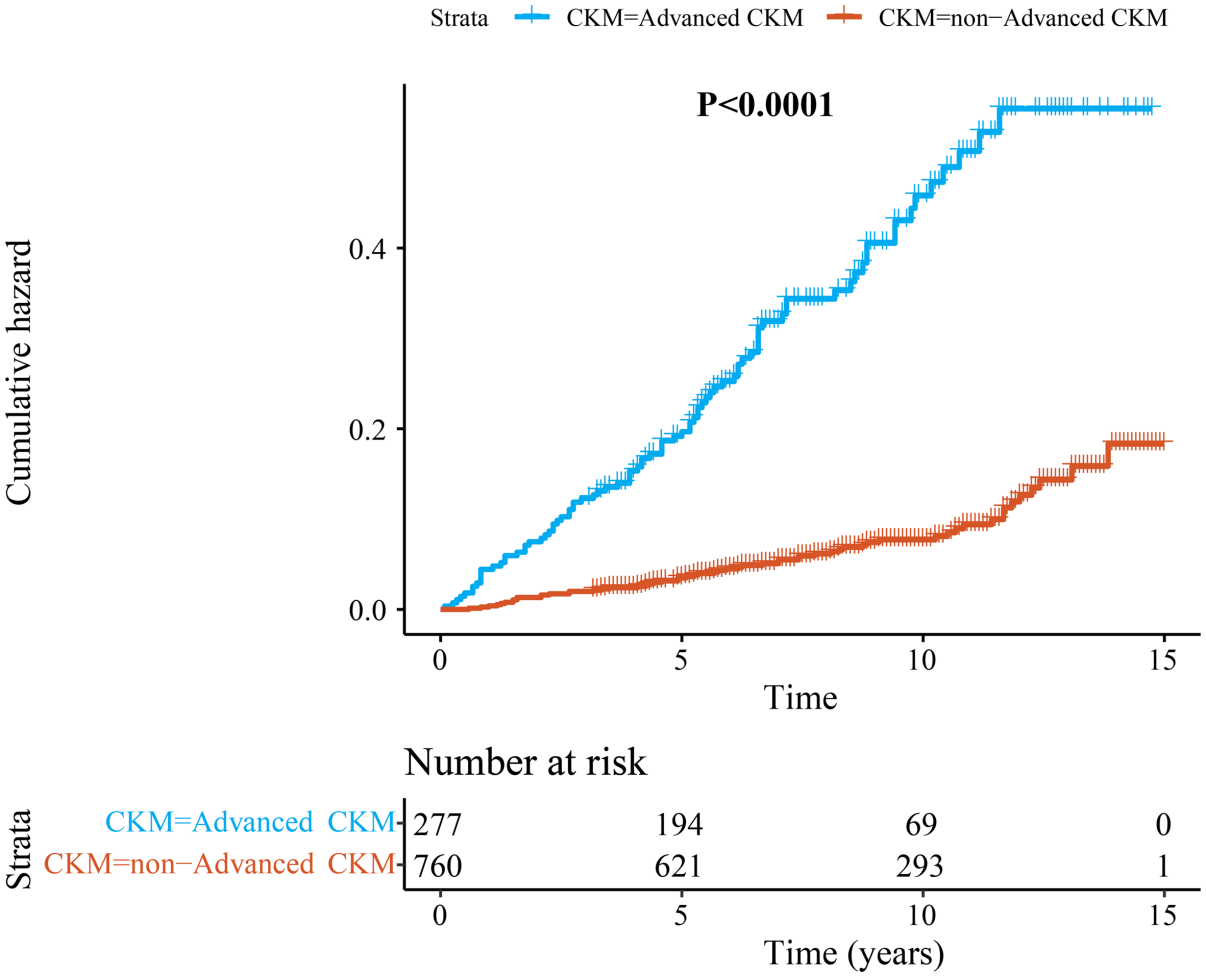
The Kaplan–Meier curves of cumulative risk for all-cause mortality among depressed patients from 1,037 participants in longitudinal cohort study. CKM syndrome; cardiovascular-kidney-metabolic syndrome.

Figure 3 shows the results of the Kaplan–Meier survival analysis. Log-rank tests were performed to show that the cumulative all-cause mortality in the advanced CKM group was significantly higher compared with the non-advanced CKM syndrome group (*p* < 0.0001).

Table 4 shows the relationship between the binary CKM syndrome and the risk of all-cause mortality among patients with depression analyzed through the Cox regression model. In comparison with the non-advanced CKM syndrome (stages 0–2) group, the risk of all-cause mortality in the advanced CKM syndrome (stages 3–4) group increased significantly in all models. Without adjustment (Model 0), advanced CKM syndrome was associated with a 3.98-fold increase in the risk of all-cause mortality (HR: 4.98, 95% CI: 3.57–6.95, *p* < 0.0001). In Model 1 where demographic and socioeconomic characteristics were adjusted, the association between advanced CKM syndrome and the risk of all-cause mortality was weakened, but it remained very significant (HR: 2.13, 95% CI: 1.45–3.13, *p* < 0.001). In Model 2 where lifestyle and BMI were further adjusted, advanced CKM syndrome was associated with a 0.99-fold increase in the risk of all-cause mortality (HR: 1.99, 95% CI: 1.33–2.96, *p* < 0.001). In Model 3 where comorbidities and medication history were fully adjusted, the risk of all-cause mortality in the advanced CKM syndrome group was 0.94 times higher than that in the non-advanced CKM syndrome group (HR: 1.94, 95% CI: 1.29–2.93, *p* = 0.002).

**Table 4:** HR estimates for the association between CKM syndrome and all-cause mortality in patients with depression from 1,037 participants in longitudinal cohort study.

		Model 0		Model 1		Model 2		Model 3	
All-cause mortality~CKM		HR (95%CI)	*P-*value	HR (95%CI)	*P-*value	HR (95%CI)	*P-*value	HR (95%CI)	*P-*value
	Non-Advanced CKM syndrome	Reference		Reference		Reference		Reference	
	Advanced CKM syndrome	4.98 (3.57, 6.95)	<0.0001	2.13 (1.45, 3.13)	<0.001	1.99 (1.33, 2.96)	<0.001	1.94 (1.29, 2.93)	0.002

Model 0: Crude model.

Model 1: Adjusted for age, sex, race, marital status, education, and poverty-income ratio.

Model 2: Additionally adjusted for drinking, smoking, total energy intake, weekly physical activity level, and body mass index.

Model 3: further adjusted for diabetes, cancer, hypertension, liver diseases, thyroid diseases, arthritis, and antidepressant.

Non-Advanced CKM syndrome was defined as CKM syndrome in stages 0–2. Advanced CKM syndrome was defined as CKM syndrome in stages 3–4. CKM syndrome, cardiovascular-kidney-metabolic syndrome; HR, hazard ratio; CI, confidence interval.

### Subgroup analysis

**Figure 4:**
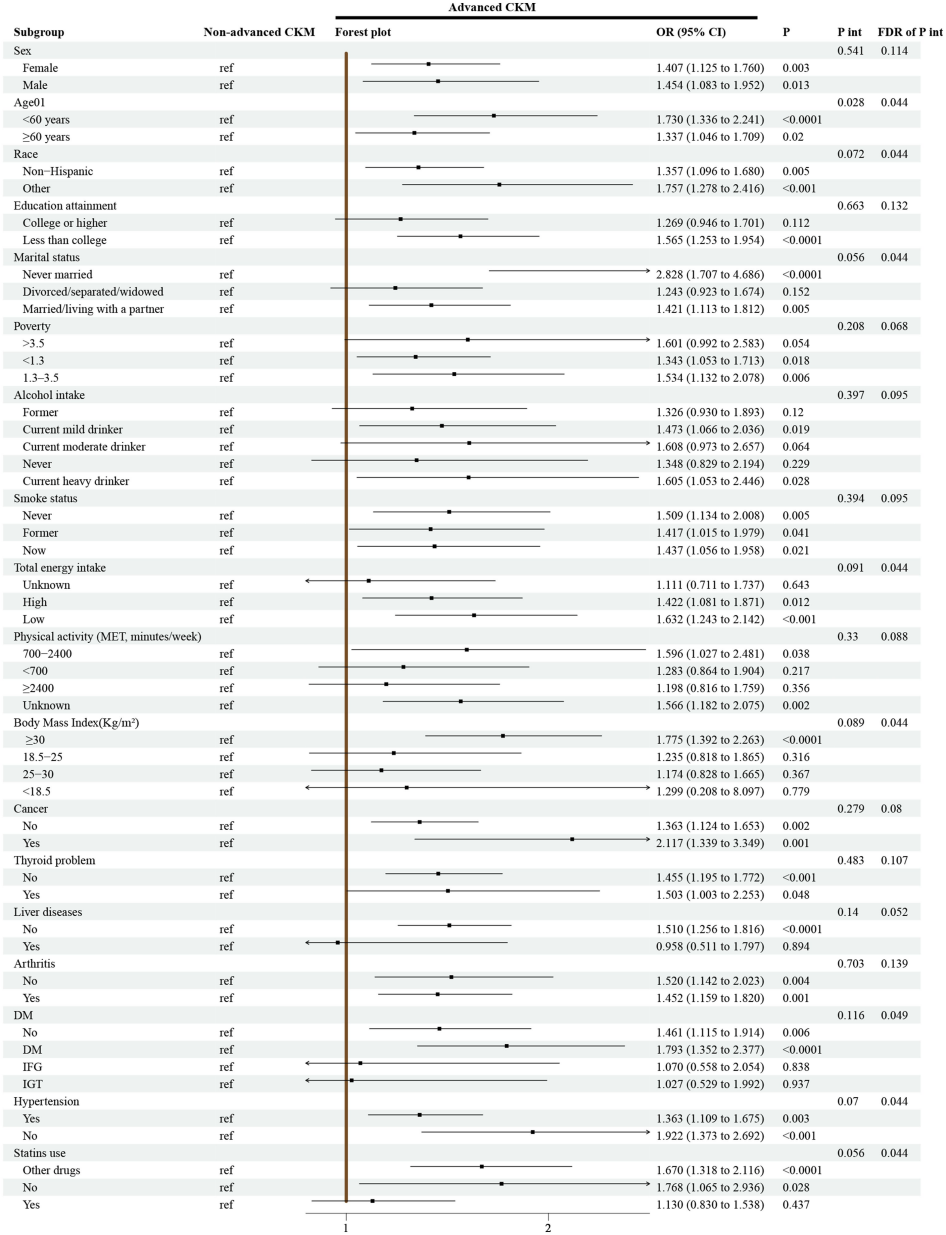
Subgroup analyses for the association between CKM syndrome and depression from 15,156 participants in cross-sectional study. Notes: Models were adjusted for age, sex, race, marital status, education, poverty-to-income ratio, drinking, smoking, total energy intake, weekly physical activity level, body mass index, diabetes, cancer, hypertension, liver diseases, thyroid diseases, arthritis, and antidepressant. CKM syndrome, cardiovascular-kidney-metabolic syndrome; MET, metabolic equivalent; DM, diabetes mellitus; IFG, impaired fasting glycaemia; IGT, impaired glucose tolerance; OR, odds ratio; CI, confidence interval; P int., P for interaction; FDR, False Discovery Rate.

Figure 4 shows the results of the subgroup analysis conducted on the association between CKM syndrome and depression. Our analysis shows that the association between CKM syndrome and depression is stronger (OR: 1.73, 95% CI: 1.34–2.24, *p* < 0.0001) in the younger subgroup (<60 years) but weaker (OR: 1.34, 95% CI: 1.05–1.71, *p* = 0.02) in the older subgroup (≥60 years). There is a significant difference between the two subgroups (P for interaction = 0.028).

**Figure 5:**
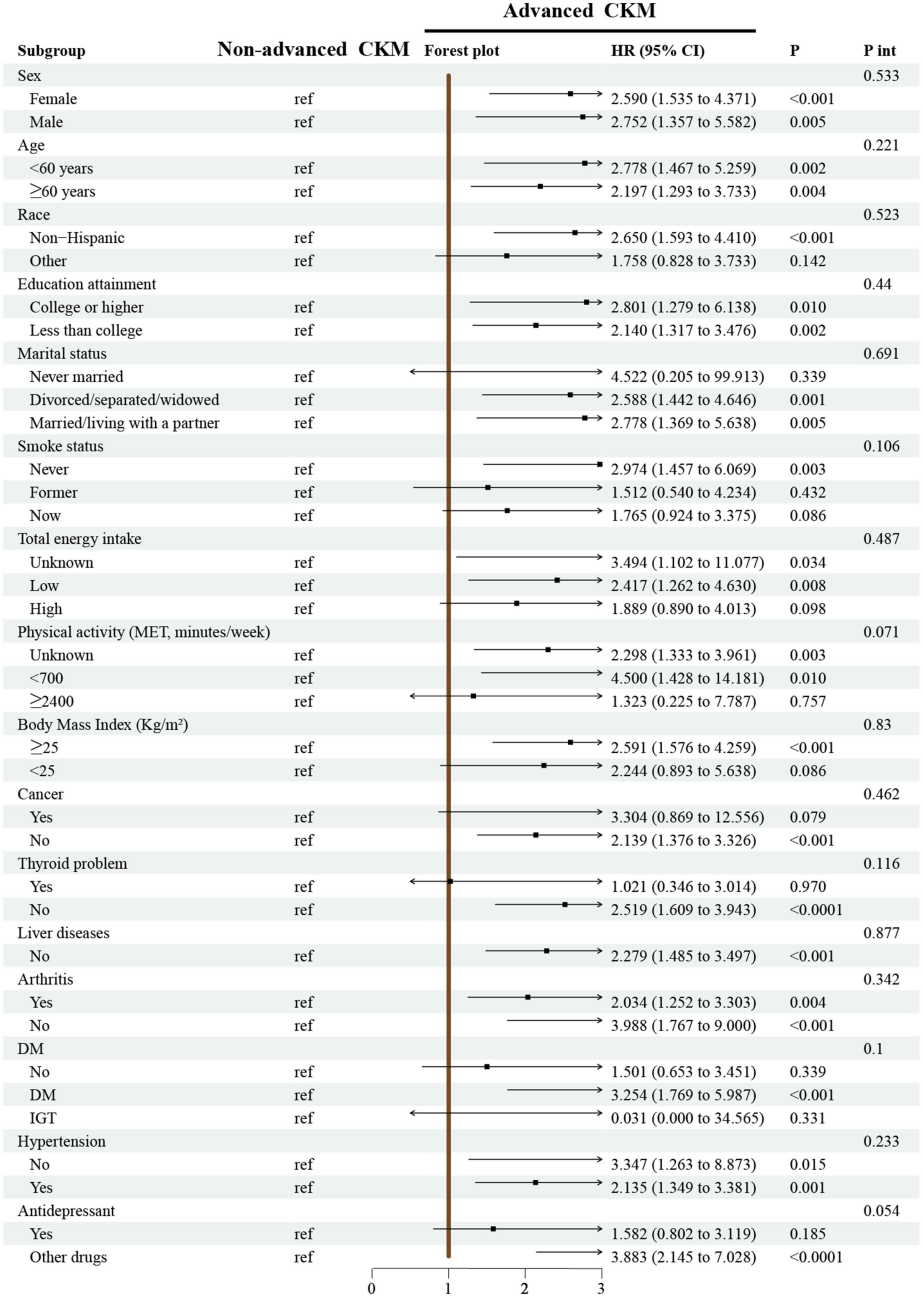
Subgroup analyses for the association between CKM syndrome and all-cause mortality in the depressed population from 1,037 participants in longitudinal cohort study. Notes: Models were adjusted for age, sex, race, marital status, education, poverty-to-income ratio, drinking, smoking, total energy intake, weekly physical activity level, body mass index, diabetes, cancer, hypertension, liver diseases, thyroid diseases, arthritis, and antidepressant. CKM syndrome, cardiovascular-kidney-metabolic syndrome; MET, metabolic equivalent; DM, diabetes mellitus; IFG, impaired fasting glycaemia; IGT, impaired glucose tolerance; HR, hazard ratio; CI, confidence interval; P int., P for interaction.

Figure 5 shows the performance of the association between CKM syndrome and the risk of all-cause mortality among patients with depression in different subgroups. Our analysis suggests a strong association between the two among different subgroups (all P for interaction > 0.05).

### Sensitivity analysis

Supplementary Table 2 presents the results of sensitivity analyses conducted on the association between CKM syndrome and depression. In the fully adjusted Model 3, weighted logistic regression indicated that the prevalence of depression in CKM stage 4 was 0.96 times higher than in CKM stage 0 (OR: 1.96, 95% CI: 1.15–3.35, *p* = 0.01). After depression was redefined, the prevalence of depression in CKM stage 4 was 0.73 times higher than in CKM stage 0 (OR: 1.73, 95% CI: 1.38–2.17, *p* < 0.0001). Following multiple imputation of covariates, the prevalence of depression in CKM stage 4 was 1.11 times higher than in CKM stage 0 (OR: 2.11, 95% CI: 1.53–2.93, *p* < 0.0001). These results support our initial findings.

Supplementary Table 3 shows the results of sensitivity analyses conducted on the risk of all-cause mortality in patients with both CKM syndrome and depression. In the fully adjusted Model 3, weighted Cox regression indicated that the risk of all-cause mortality in the advanced CKM syndrome group was 0.82 times higher than in the non-advanced CKM syndrome group (HR: 1.82, 95% CI: 1.18–2.81, *p* = 0.01). After depression was redefined, the risk of all-cause mortality in the advanced CKM syndrome group was 0.71 times higher than in the non-advanced CKM syndrome group (HR: 1.71, 95% CI: 1.33–2.19, *p* < 0.0001). Following multiple imputation, the risk of all-cause mortality in the advanced CKM syndrome group was 1.14 times higher than in the non-advanced CKM syndrome group (HR: 2.14, 95% CI: 1.47–3.12, *p* < 0.0001). These results further verify the robustness of this study.

## Discussion

In this large-scale observational study involving 15,156 participants, the prevalence of depression in different stages of CKM syndrome was first investigated. The results showed that only the prevalence of depression in stage 4 of CKM syndrome was higher than in the normal population (stage 0 of CKM syndrome) after confounding factors were fully adjusted. Moreover, the prevalence of depression in the advanced CKM syndrome (stages 3–4) group was 47% higher than in the non-advanced CKM syndrome group (stages 0–2). This association was more evident in the younger subgroup (<60 years). In addition, a longitudinal survival cohort was constructed in this study by linking the NHANES database with the NDI database with follow-up until December 31, 2019. During survival analysis, advanced CKM syndrome was associated with a 94% increase in the risk of all-cause mortality among patients with depression. In summary, the relationship between CKM syndrome under the latest definition, depression, and the risk of all-cause mortality was elucidated in this study.

In the US population, the relationship between CKM syndrome, depression, and all-cause mortality remains unelucidated. Previously, Huang et al. (24), based on the UK population, found a positive association between CKM syndrome staging and depression. Our study extends this conclusion to the US population. Different from our research purpose, Li et al. (12) investigated 13,396 participants from NHANES and found that the severity of depressive symptoms was associated with all-cause mortality across all CKM stages. Our study emphasizes that in the depressed population, CKM syndrome increases the risk of all-cause mortality. In addition, considering that CKM syndrome may be comorbid with depression, two studies from the United States and China, respectively, developed prediction models for depression in patients with CKM syndrome, and they consistently found that gender is an important predictor (25, 26).

The definitions of stages 0–4 of CKM syndrome are as follows. Stage 0 is determined when both the BMI and waist circumference are within the normal range, with the participants meeting this condition not included in higher stages. Once a participant shows an increase in BMI, an increase in waist circumference, or the features of prediabetes, he or she is classified into stage 1. When a participant has metabolic-related risk factors or is at a moderate or high risk of developing CKD, he or she is classified into stage 2. The criterion for defining stage 3 is that the participant has a high risk of CKD or is predicted to have a high risk of developing CVD according to the 10-year CVD prediction model. If a participant has already suffered from CVD, then he or she is classified into stage 4 (11). The current study shows that there is a trend effect among the four groups when stage 0 is taken as a reference, with OR values of 1.06, 1.25, 1.38, and 1.98 respectively, although only the prevalence rate of depression in the CKM syndrome group of stage 4 is significantly higher. It is indicated that the prevalence rate of depression gradually increases with the progression of CKM syndrome. Previous studies have shown that obesity, diabetes, CKD, and CVD are risk factors for depression. Earlier, a meta-analysis showed that being overweight increased the risk of depressive episodes during follow-up (27). Additionally, a population-based study suggested that compared with the first quartile group of the weight-adjusted waist index (WWI), only the participants in the fourth quartile group had an increased risk of depression, underscoring the high risk of depression among potential obese individuals (28). Although diabetes is also a risk factor for depression, some studies have demonstrated that there is no significant correlation between PHQ-9 and prediabetes after non-glucose factors are adjusted (29). In this study, a participant is classified into stage 1 of CKM syndrome if he or she shows an increase in BMI, an increase in waist circumference, or the features of prediabetes. Therefore, it is suspected that the non-significant increase in the risk of depression in stage 1 of CKM syndrome compared with stage 0 observed in this study is the combined result of obesity and prediabetes. Metabolism-related risk factors are also risk factors for depression. According to Meng et al. (30), metabolic syndrome (MetS) and its components, including central obesity, hypertension, hyperglycemia, and dyslipidemia, are positively correlated with the PHQ-9 score. As for stage 3 of CKM syndrome, it still showed no significantly higher risk of depression, which is consistent with the study of Ricardo et al. (31), where the presence of CKD shows no significant correlation with depressive symptoms in the logistic regression analysis. Having already suffered from CVD is defined as stage 4 of CKM syndrome, and the current study shows that the prevalence rate of depression in participants of stage 4 increased by 98% compared with that of stage 0. Both CVD and depression are common, and patients with CVD are more likely to develop depression than the general population (32). For example, among patients considered disabled after a myocardial infarction, 40% of them have depression (32), and two-thirds of consecutive patients develop depressive and anxious symptoms after being admitted to the hospital due to a cardiac event (33). In conclusion, the results of this study suggest that the risk of developing depression becomes increasingly higher as CKM syndrome deteriorates. It is worth noting that this study also seems to imply the priority of CVD and CKD over metabolic abnormalities, because the risk of depression in advanced CKM syndrome increases significantly by 47% compared with that in CKM syndrome of stages 0–2 when stages 3 and 4 of CKM syndrome are defined as the advanced stages. This finding suggests that timely identification and prevention of the deterioration of CKM syndrome may help reduce the possibility of depression.

Studies have shown that patients with depression have a significantly higher risk of all-cause mortality compared to those without depressive symptoms (34). Depression not only increases the risk of death alone but also acts in conjunction with other diseases such as diabetes, cardiovascular diseases, and chronic diseases, significantly increasing the risk of all-cause mortality. For example, among patients with diabetes, depression increases the risk of all-cause mortality by 1.63 times (35). In patients with coronary heart disease, depression has been confirmed as the strongest predictor of all-cause mortality, with a risk even exceeding that of diabetes, smoking, and hypertension (36). In addition, the interaction between depression and chronic diseases also leads to a significant increase in the risk of all-cause mortality. For instance, the risk of all-cause mortality in patients with mild depression is 1.25 times higher than in those without chronic diseases, and it is 2.03 times higher in patients with severe depression (37). Moreover, patients with CVD are more likely to develop depression than the general population. Patients with depression are ultimately more likely to develop CVD and have a higher mortality rate than the general population. CVD patients with depression have a worse prognosis than those without depression (32). However, it remains inconclusive whether the risk of all-cause mortality is higher in patients with depression who have CKM syndrome. The current study further expands the knowledge in this field, as CKM syndrome also interacts with depression. Compared with the participants in stages 0–2 of CKM syndrome, advanced CKM syndrome (stages 3–4) increases the risk of all-cause mortality by 94%.

The subgroup analysis reveals a stronger association between CKM syndrome and depression in the younger subgroup (<60 years). Firstly, the physical metabolism and physiological functions of the younger population are relatively more active. The impacts of metabolic disorders, inflammatory responses, etc., involved in CKM syndrome, on the body’s internal environment may be more significant, making it easier to trigger depressive moods by affecting the neurotransmitter system, neuroendocrine system, etc. For example, the inflammatory response caused by CKM syndrome may interfere with the normal metabolism and transmission of neurotransmitters such as serotonin and dopamine. Since the nervous system of the younger population may be more sensitive to these changes, depressive symptoms are more likely to occur (38, 39). Secondly, the cardiovascular system and renal function reserve of the younger population are relatively better (40). It is speculated that younger individuals are more likely to compensate for the abnormal changes in the body at the early stage of CKM syndrome, making some physical symptoms less obvious. However, they may be more sensitive psychologically to the stress caused by the disease, changes in lifestyle, etc., which makes them more prone to depressive moods. Also, this kind of mood may, to a certain extent, exacerbate the body’s stress response, forming a vicious cycle and strengthening the association between the two. Finally, compared with the elderly population, the social roles of the younger population are more complex and diverse. After getting sick, they may face more pressure from different social roles, such as worrying about being discriminated against at work and being a burden for the family. Without effective social support, these pressures may exacerbate depressive moods, as a result of which a stronger association is manifested between CKM syndrome and depression.

Based on the stronger association between CKM syndrome and depression in younger populations, future research could be expanded across multiple dimensions. Firstly, at the mechanistic level, studies could be conducted by measuring dynamic changes in inflammatory cytokines and neurotransmitters to validate the specific role of the “metabolic dysregulation-neural regulation abnormality” pathway in younger cohorts. Integrating qualitative and quantitative approaches would be conducive to exploring the correlation between social role stress, psychological resilience, and disease progression. Secondly, randomized controlled trials should be conducted to evaluate the efficacy of combined interventions (i.e., metabolic management plus psychological counseling) in reducing depression risk among young CKM patients. Concurrently, it is necessary to promote early identification by developing the risk prediction models where CKM-specific biomarkers are integrated with psychosocial factors. Thirdly, cross-population studies across diverse ethnic and geographic groups are required to verify whether the observed association varies by cultural context or healthcare accessibility. Comparative analyses with other chronic diseases would help further illuminate the unique contributions of CKM syndrome to depression. These directions will lay a more robust theoretical and practical foundation for the precision prevention and treatment of depression in young individuals with CKM syndrome.

This study emphasizes that the CKM syndrome staging system can assist clinicians in identifying patients at different disease risk stages earlier, especially those individuals who have not yet shown obvious clinical manifestations of cardiovascular or renal diseases but already have early warning signs such as metabolic abnormalities. For example, for patients in stage 1 of CKM syndrome, although they may not have developed into confirmed chronic kidney disease or cardiovascular disease yet, doctors can pay attention to their metabolic disorders according to the prompts of the staging system. Meanwhile, timely lifestyle interventions or necessary drug treatments can be provided to control body weight, improve metabolic indicators such as blood glucose and blood lipids, and delay the progression of the disease to more severe stages, thereby reducing the risk of subsequent depression and all-cause mortality, which reflects the practical value of the staging system in the early prevention and control of diseases.

The stages of CKM syndrome correspond to different treatment focuses and strategies, providing a basis for personalized medicine. In the early stages (such as stages 1–2), the emphasis may be placed on improving the overall metabolic status through lifestyle changes and metabolic-regulating drugs. In the middle and late stages (such as stages 3–4), in addition to continuing to control metabolic factors, targeted treatments for the existing renal or cardiovascular problems are also required. At the same time, attention should be paid to the patients’ mental health status, and timely comprehensive intervention measures such as psychological support or antidepressant treatment should be provided. Through actual cases, the good results achieved by patients in disease control, improvement of mental health, and enhancement of quality of life after formulating personalized treatment plans according to the staging system are demonstrated, highlighting the important role of this staging system in clinical treatment decisions.

It should be noted that as a commonly used depression screening tool, the PHQ-9 only captures symptoms within the preceding two weeks, which may result in temporal limitations in the assessment of chronic depressive states (17). If depressive symptoms in patients with CKM syndrome exhibit a chronic persistent pattern, this tool may underestimate the actual prevalence of depression, thereby potentially weakening the strength of the long-term association between CKM syndrome and depression. Meanwhile, patients with CKM syndrome are often accompanied by multisystem somatic symptoms, which may include pain, fatigue, and sleep disturbances. These symptoms overlap with some assessment items of the PHQ-9, which can lead to a spurious increase in scores. Particularly in patients with advanced CKM syndrome, such interference from somatic symptoms may increase the risk of misclassifying non-depressive states as depression, introducing a certain degree of misclassification bias (17, 41). On the other hand, compared with the potential inadequacy of traditional diagnostic classifications for depression in populations with complex comorbid physical illnesses, the PHQ-9, by quantifying the severity of symptoms, is better able to reflect fluctuations in emotional symptoms associated with the pathophysiological processes of CKM syndrome. Instead, it may be more aligned with the actual clinical manifestations of this specific population (42, 43). The aforementioned factors suggest that when interpreting the results of this study, caution should be exercised to distinguish between the contributions of CKM-related somatic symptoms and primary depressive symptoms. The “depression” assessed by the PHQ-9 is more inclined to reflect an association at the clinical symptom dimension, rather than an association in the strict diagnostic sense. This also provides a direction for subsequent studies to conduct validation using tools such as the Structured Clinical Interview for DSM (SCID), in order to further clarify the core mechanism underlying the true pathological association between CKM syndrome and depression.

The CKM staging framework breaks through the limitations of traditional single-organ disease assessment by integrating the cardiovascular system, renal function, and metabolic status into an interconnected whole for the first time, which accurately aligns with the pathophysiological linkage mechanism of the “cardio-renal-metabolic” axis. Notably, the cumulative effect of such multisystem disorders is closely associated with the risk of depression. Compared with traditional frameworks that focus solely on individual diseases, the CKM staging system can more comprehensively capture the synergistic impacts of metabolic abnormalities and cardio-renal injuries on the neurotransmitter system and inflammatory pathways. It is particularly suitable for identifying high-risk populations with potential depressive risks due to the accumulation of early multidimensional risks, thereby providing more precise upstream intervention targets for the simultaneous prevention and control of physical diseases and mental health issues. In addition, the hierarchical characteristics of CKM can offer a basis for differentiated clinical interventions: in the early stages (Stages 0–2) of CKM, depression triggers can be reduced by regulating metabolic indicators and improving lifestyle; in the middle and advanced stages (Stages 3–4), targeted psychological screening and intervention can be implemented while strengthening cardio-renal protection, achieving the coordinated management of physical and mental health. Regarding the priorities for future research in this field, we propose three key directions: first, conduct multicenter prospective cohort studies to systematically verify the strength of the association between each CKM stage and the incidence, recurrence rate, and long-term adverse outcomes of depression, further clarifying the dose–response relationship between staging and depressive risk; second, integrate multi-omics technologies to screen specific biomarkers associated with both CKM stage progression and depression onset, developing precise tools with dual early warning functions for physical and mental risks; third, evaluate the efficacy of individualized intervention strategies tailored to different CKM stages in reducing depressive risk and improving patients’ overall prognosis through randomized controlled trials (RCTs), clarify the practical application value of this framework in integrating the prevention and treatment of cardio-renal-metabolic diseases and depression, and ultimately form a standardized comprehensive management pathway.

### Advantages

In this study, the association between the newly defined CKM syndrome and depression was analyzed to systematically elucidate the prevalence of depression among different stages of CKM syndrome. Also, CKM syndrome was redefined as early and late stages to demonstrate that even maintaining CKM syndrome at stages 0–2 may still bring benefits and reduce the incidence of depression. Importantly, it was also revealed in this study that CKM syndrome increases the long-term mortality risk of patients with depression, which highlights the significant role of maintaining good cardiovascular and renal functions in improving the long-term quality of life of patients with depression.

### Limitations

However, there remain some limitations worthy of consideration. Firstly, there are non-negligible limitations in this study regarding the revelation of the association between CKM syndrome and depression. Since this study is based on a cross-sectional study design, it only captures the associated state of the two at the same moment and fails to determine the causal sequence. Specifically, we are unclear whether it is the advanced stage of CKM syndrome that induces depression or whether depression, as an influencing factor, further exacerbates the progression of CKM syndrome. The ambiguity of this causal relationship constitutes the core problem that the research findings cannot effectively guide clinical actions or policy-making. In clinical practice, medical staff expect to formulate personalized treatment plans based on clear causal associations, such as intervening in the key pathogenic links in the causal relationship. In terms of policy-making, clear causal information helps to determine the focus of resource allocation and the direction of preventive interventions. However, the current study is limited by the cross-sectional design and cannot provide such crucial information. Future studies can conduct longitudinal cohort studies with long-term follow-up to dynamically observe patients, thereby determining the causal relationship between the two and enhancing the guiding role of the research on practice and policy. Secondly, American adults were analyzed in this study, and the conclusion is not applicable to be generalized to other populations. Thirdly, some potential confounding factors may be inevitably overlooked. Fourthly, in the NHANES database, the lack of clinical examination evidence such as echocardiography may cause the prevalence of advanced CKM syndrome to be underestimated (8). Finally, most of the diagnostic items for depression are based on self-report, which is affected by recall bias.

## Conclusion

This study emphasizes the associations among CKM syndrome defined by the latest AHA criteria, depression, and mortality. Stage 4 of CKM syndrome increases the likelihood of depression by 98%, and advanced CKM syndrome increases the prevalence of depression by 47%. Additionally, advanced CKM syndrome reduces the long-term quality of survival of patients with depression. These findings indicate the importance of paying attention to the progression of CKM syndrome for the prevention of depression and its prognosis.

## Data Availability

Publicly available datasets were analyzed in this study. This data can be found at: the website for cross-sectional data is https://wwwn.cdc.gov/nchs/nhanes/; the website for survival data is https://www.cdc.gov/nchs/ndi/?CDC_AAref_Val=https://www.cdc.gov/nchs/ndi/index.htm.

## Supplementary material

The Supplementary material for this article can be found online at:

https://www.frontiersin.org/articles/10.3389/fnut.2025.1620008/full#supplementary-material

## Glossary

AHA: American Heart Association
BMI: Body mass index
CI: Confidence interval
CKD: Chronic kidney disease
CKM: Cardiovascular-Kidney-Metabolic Syndrome
CVD: Cardiovascular diseases
DM: Diabetes mellitus
HR: Hazard ratio
IQR: Interquartile range
KDIGO: Kidney Disease Improving Global Outcomes
MetS: Metabolic syndrome
NDI: National Death Index
NHANES: National Health and Nutrition Examination Survey
OR: Odds ratio
PHQ-9: Patient Health Questionnaire - 9
PIR: Poverty income ratio
PREVENT: Predicting Risk of CVD EVENTs
RCS: Restricted cubic spline
RCTs: Randomized controlled trials
VIF: Variance inflation factor
WHO: World Health Organization
WWI: Weight-adjusted waist index
