# Supplementary material for: Association of cardiovascular-kidney-metabolic syndrome with depression and all-cause mortality: a population-based observational study
Source: Front Nutr. 2025 Nov 3;12:1620008. doi: 10.3389/fnut.2025.1620008 (PMC12620474; doi:10.3389/fnut.2025.1620008)
Supplement: Supplementary file 1 [file Table_1.docx]

**Association of cardiovascular-kidney-metabolic syndrome with depression and all-cause mortality: A population-based observational study**

Wei Liu^1*^, Zhijuan Li^1^, Pu Ma^1^

^1^ Department of Cardiovascular, The First Affiliated Hospital, and College of Clinical Medicine of Henan University of Science and Technology, Luoyang, China.

^*^ Correspondence author: Wei Liu
Email: [wei_liu1980@163.com](mailto:wei_liu1980@163.com)

## Supplementaty Table 1: Definition of cardiovascular-kidney-metabolic syndrome stages:

| Definition the stages of CKM syndrome | |  |
| --- | --- | --- |
| CKM Stage 0: | CKM Stage 0 included individuals with a normal body mass index (BMI) (less than 23 kg/m² for those of Asian ethnicity and less than 25 kg/m² for other racial and ethnic groups) and a normal waist circumference (less than 80 cm for Asian women and less than 90 cm for Asian men, while less than 88 cm for women and less than 102 cm for men in all other racial and ethnic groups), who did not meet the criteria for any of the other stages. |  |
|  |  |  |
|  |  |  |
|  |  |  |
| CKM Stage 1: | CKM Stage 1 included individuals with either an elevated BMI (≥23 kg/m² for those of Asian ethnicity and >25 kg/m² for other racial and ethnic groups), an increased waist circumference (≥80 cm for Asian women and ≥90 cm for Asian men, and ≥88 cm for women and ≥102 cm for men in other racial and ethnic groups), or prediabetes, defined as having glycated hemoglobin levels between 5.7% and <6.5%, or fasting blood glucose levels between 100 mg/dL and <126 mg/dL. |  |
|  |  |  |
|  |  |  |
|  |  |  |
| CKM Stage 2: | CKM Stage 2 included individuals with metabolic risk factors or moderate-to-high-risk chronic kidney disease (CKD) as defined by Kidney Disease Improving Global Outcomes (KDIGO) criteria, in line with AHA recommendations. The qualifying metabolic risk factors were elevated fasting serum triglycerides (≥135 mg/dL), hypertension, diabetes, or metabolic syndrome, which was defined as having at least 3 of the following: increased waist circumference, low high-density lipoprotein (HDL) cholesterol (<40 mg/dL for men or <50 mg/dL for women), fasting serum triglycerides ≥150 mg/dL, elevated blood pressure (systolic blood pressure ≥130 mmHg, diastolic blood pressure ≥80 mmHg, and/or the use of blood pressure-lowering medications), or prediabetes. CKD stages were determined based on glomerular filtration rate (GFR) and the urinary albumin-to-creatinine ratio. |  |
|  |  |  |
|  |  |  |
|  |  |  |
| CKM Stage 3: | CKM Stage 3 was determined by the presence of very-high-risk CKD according to KDIGO stages or a high predicted 10-year cardiovascular disease (CVD) risk. The 10-year CVD risk was estimated using the AHA's Predicting Risk of CVD Events (PREVENT) equations, with high risk defined as a 10-year CVD risk of ≥20%.(Khan et al., 2024) The PREVENT equations were specifically developed and validated for adults aged 30 to 79.As such, risk was not estimated for adults <30 years. However, to minimize underestimation of CKD Stage 3, adults ≥80 years were not excluded from 10-year CVD risk. Instead, adults ≥80 years were assigned an age of 79 years when determining 10-year  CVD risk to allow for conservative estimates. Further, PREVENT was developed for variables with the following ranges: total cholesterol 130-320 mg/dL, HDL 20-100 mg/dL, systolic blood pressure 90-200 mmHg, and GFR 14-140 mL/min/1.73m². To approximate PREVENT risk strata, values for these variables above or below these bounds were set to the upper or lower bounds of allowable values respectively (for example, total cholesterol of 330 mg/dL was set as 320 mg/dL). Cardiac biomarkers and cardiovascular imaging were not available to identify subclinical CVD. |  |
|  |  |  |
|  |  |  |
|  |  |  |
| CKM Stage 4: | CKM Stage 4 was identified based on self-reported established cardiovascular disease (coronary heart disease, angina, heart attack, heart failure, and stroke). Atrial fibrillation and peripheral artery disease were excluded due to the unavailability of relevant data. |  |
|  |  |  |
|  |  |  |
|  |  |  |

## Supplementaty Table 2: OR estimates for the association between CKM syndrome and depression in several sensitivity analyses

|  | **Model 0** | | **Model 1** | | **Model 2** | | **Model 3** | |
| --- | --- | --- | --- | --- | --- | --- | --- | --- |
|  | **OR (95%CI)** | ***P-*value** | **OR (95%CI)** | ***P-*value** | **OR (95%CI)** | ***P-*value** | **OR (95%CI)** | ***P-*value** |
| **^1^Weighted logistic regression** |  |  |  |  |  |  |  |  |
| **CKM 0** | **reference** |  | **reference** |  | **reference** |  | **reference** |  |
| **CKM 1** | 1.38(0.94,2.03) | 0.10 | 1.56(0.96,2.51) | 0.07 | 1.26(0.71,2.22) | 0.42 | 1.27(0.72,2.24) | 0.40 |
| **CKM 2** | 1.82(1.28,2.58) | <0.001 | 2.10(1.44,3.08) | <0.001 | 1.73(1.06,2.81) | 0.03 | 1.51(0.93,2.44) | 0.09 |
| **CKM 3** | 1.36(0.92,2.02) | 0.13 | 1.49(1.00,2.22) | 0.05 | 1.32(0.80,2.18) | 0.28 | 1.57(0.95,2.60) | 0.08 |
| **CKM 4** | 3.45(2.34,5.10) | <0.0001 | 3.91(2.53,6.05) | <0.0001 | 2.92(1.71,4.98) | <0.001 | 1.96(1.15,3.35) | 0.01 |
| **p for trend** |  | <0.0001 |  | <0.0001 |  | <0.001 |  | 0.01 |
|  |  |  |  |  |  |  |  |  |
| **Non-Advanced CKM syndrome** | **reference** |  | **reference** |  | **reference** |  | **reference** |  |
| **Advanced CKM syndrome** | 1.79(1.49,2.16) | <0.0001 | 1.66(1.35,2.06) | <0.0001 | 1.51(1.22,1.87) | <0.001 | 1.28(1.02,1.62) | 0.03 |
| **^2^Redefined depression** |  |  |  |  |  |  |  |  |
| **CKM 0** | **reference** |  | **reference** |  | **reference** |  | **reference** |  |
| **CKM 1** | 1.05(0.89,1.23) | 0.57 | 1.16(0.98,1.37) | 0.08 | 0.88(0.73,1.06) | 0.17 | 0.99(0.82,1.19) | 0.88 |
| **CKM 2** | 1.36(1.18,1.57) | <0.0001 | 1.63(1.40,1.91) | <0.0001 | 1.16(0.98,1.39) | 0.09 | 1.09(0.91,1.31) | 0.37 |
| **CKM 3** | 1.19(0.97,1.48) | 0.10 | 1.77(1.38,2.26) | <0.0001 | 1.28(0.99,1.66) | 0.06 | 1.25(0.95,1.63) | 0.11 |
| **CKM 4** | 2.41(2.04,2.85) | <0.0001 | 3.24(2.67,3.94) | <0.0001 | 2.20(1.78,2.73) | <0.0001 | 1.73(1.38,2.17) | <0.0001 |
| **p for trend** |  | <0.0001 |  | <0.0001 |  | <0.0001 |  | <0.001 |
|  |  |  |  |  |  |  |  |  |
| **Non-Advanced CKM syndrome** | **reference** |  | **reference** |  | **reference** |  | **reference** |  |
| **Advanced CKM syndrome** | 1.57(1.43,1.72) | <0.0001 | 1.76(1.57,1.97) | <0.0001 | 1.69(1.50,1.90) | <0.0001 | 1.46(1.29,1.66) | <0.0001 |
| **^3^Multiple imputation** |  |  |  |  |  |  |  |  |
| **CKM 0** | **reference** |  | **reference** |  | **reference** |  | **reference** |  |
| **CKM 1** | 1.05(0.81,1.37) | 0.71 | 1.15(0.88,1.50) | 0.31 | 1.03(0.77,1.38) | 0.84 | 1.13(0.84,1.52) | 0.42 |
| **CKM 2** | 1.59(1.27,2.01) | <0.0001 | 1.88(1.49,2.41) | <0.0001 | 1.49(1.15,1.96) | 0.003 | 1.34(1.02,1.78) | 0.04 |
| **CKM 3** | 1.34(0.97,1.83) | 0.07 | 1.96(1.36,2.80) | <0.001 | 1.54(1.05,2.24) | 0.03 | 1.47(0.99,2.18) | 0.06 |
| **CKM 4** | 3.17(2.49,4.08) | <0.0001 | 4.07(3.09,5.41) | <0.0001 | 3.04(2.24,4.14) | <0.0001 | 2.11(1.53,2.93) | <0.0001 |
| **p for trend** |  | <0.0001 |  | <0.0001 |  | <0.0001 |  | <0.0001 |
|  |  |  |  |  |  |  |  |  |
| **Non-Advanced CKM syndrome** | **reference** |  | **reference** |  | **reference** |  | **reference** |  |
| **Advanced CKM syndrome** | 1.80(1.60,2.03) | <0.0001 | 1.96(1.69,2.27) | <0.0001 | 1.83(1.58,2.12) | <0.0001 | 1.47(1.25,1.72) | <0.0001 |

Notes:

Model 0: Crude model.

Model 1: Adjusted for age, sex, race, marital status, education, and poverty-income ratio.

Model 2: Additionally adjusted for drinking, smoking, total energy intake, weekly physical activity level, and body mass index.

Model 3: further adjusted for diabetes, cancer, hypertension, liver diseases, thyroid diseases, arthritis, and antidepressant.

Non-Advanced CKM syndrome was defined as CKM syndrome in stages 0-2. Advanced CKM syndrome was defined as CKM syndrome in stages 3-4. CKM syndrome, cardiovascular-kidney-metabolic syndrome; OR, odds ratio; CI, confidence interval.

^1^**Weighted logistic regression was used, and all models accounted for sample weights.**

**^2^Depression was redefined with a PHQ-9 cutoff of 5.**

**^3^Multiple imputation was performed to handle missing covariates.**

## Supplementaty Table 3: HR estimates for the association between CKM syndrome and all-cause mortality among patients with depression in several sensitivity analyses

| **All-cause mortality~CKM** |  | **Model 0** | | **Model 1** | | **Model 2** | | **Model 3** | |
| --- | --- | --- | --- | --- | --- | --- | --- | --- | --- |
| **^1^Weighted Cox regression** |  | **HR (95%CI)** | ***P-*value** | **HR (95%CI)** | ***P-*value** | **HR (95%CI)** | ***P-*value** | **HR (95%CI)** | ***P-*value** |
|  | **non-Advanced CKM syndrome** | **reference** |  | **reference** |  | **reference** |  | **reference** |  |
|  | **Advanced CKM syndrome** | 5.10(3.32,7.84) | <0.0001 | 2.01(1.35,2.99) | <0.001 | 1.82(1.16,2.85) | 0.01 | 1.82(1.18,2.81) | 0.01 |
| **^2^Redefined depression** |  |  |  |  |  |  |  |  |  |
|  | **non-Advanced CKM syndrome** | **reference** |  | **reference** |  | **reference** |  | **reference** |  |
|  | **Advanced CKM syndrome** | 6.65(5.44,8.12) | <0.0001 | 1.88(1.48,2.39) | <0.0001 | 1.78(1.40,2.27) | <0.0001 | 1.71(1.33,2.19) | <0.0001 |
| **^3^Multiple imputation** |  |  |  |  |  |  |  |  |  |
|  | **non-Advanced CKM syndrome** | **reference** |  | **reference** |  | **reference** |  | **reference** |  |
|  | **Advanced CKM syndrome** | 5.77(4.22,7.88) | <0.0001 | 2.50(1.75,3.57) | <0.0001 | 2.44(1.69,3.52) | <0.0001 | 2.14(1.47,3.12) | <0.0001 |

Notes:

Model 0: Crude model.

Model 1: Adjusted for age, sex, race, marital status, education, and poverty-income ratio.

Model 2: Additionally adjusted for drinking, smoking, total energy intake, weekly physical activity level, and body mass index.

Model 3: further adjusted for diabetes, cancer, hypertension, liver diseases, thyroid diseases, arthritis, and antidepressant.

Non-Advanced CKM syndrome was defined as CKM syndrome in stages 0-2. Advanced CKM syndrome was defined as CKM syndrome in stages 3-4. CKM syndrome, cardiovascular-kidney-metabolic syndrome; HR, hazard ratio; CI, confidence interval.

^1^**Weighted logistic regression was used, and all models accounted for sample weights.**

**^2^Depression was redefined with a PHQ-9 cutoff of 5.**

**^3^Multiple imputation was performed to handle missing covariates.**
